# Sensory Saturation (3T) for Pain Management During ROP Screening in Preterm Infants Born Between 32 to 36 Weeks of Gestational Age

**DOI:** 10.1155/joph/5427050

**Published:** 2026-06-03

**Authors:** Yongli Ni, Xuejun Zhao, Jiamin Xu, Chaoqun Zhou

**Affiliations:** ^1^ Department of Neonatology, The Affiliated Yangming Hospital of Ningbo University, Ningbo, China

**Keywords:** pain management, preterm infants, retinopathy of prematurity, sensory saturation

## Abstract

**Objective:**

This study aimed to assess the effectiveness of the 3T sensory saturation method—taste, auditory, and tactile stimulation—in reducing pain and enhancing comfort in preterm infants undergoing retinopathy of prematurity (ROP) screening, compared with conventional pacifier use.

**Methods:**

A total of 100 preterm infants (gestational age 32–36 weeks) were prospectively enrolled and randomly assigned using computer‐generated random numbers into a 3T group (*n* = 52) or control group (*n* = 48). The 3T group received oral glucose, maternal voice recording, and gentle massage before and during screening, while the control group received pacifiers. Pain and comfort were evaluated using the neonatal infant pain scale and preterm infant comfort scale, alongside measurements of heart rate and oxygen saturation. Outcome assessors were blinded to group allocation.

**Results:**

Infants in the 3T group showed significantly higher oxygen saturation and lower pain scores during screening compared with controls (*p* < 0.0001). Comfort scale scores were also significantly better in the 3T group across multiple domains, including vigilance, calmness, respiratory status, physical activity, muscle tone, and mean heart rate. Multivariate linear regression identified pre‐examination vigilance, physical activity, muscle tone, facial expression, and mean heart rate as significant predictors of comfort during screening. Firth penalized logistic regression confirmed these findings with clinically reasonable odds ratios. The predictive model demonstrated strong discrimination (AUC = 0.994).

**Conclusion:**

The 3T sensory saturation method is an effective, safe, and practical nonpharmacological intervention for reducing pain and improving comfort during ROP screening in preterm infants born between 32 and 36 weeks of gestational age. These findings may not be directly generalizable to extremely preterm infants (< 32 weeks), who represent a distinct and more vulnerable population commonly included in ROP screening programs in many countries. Its adoption in clinical practice could enhance procedural tolerance and overall care for this vulnerable population.

**Trial Registration:** Chinese Registry of Clinical Trials: ChiCTR2500110779

## 1. Introduction

Retinopathy of prematurity (ROP) is a significant cause of blindness in preterm infants, necessitating regular screenings for early intervention [1]. These procedures can be painful, potentially affecting neurodevelopment and physiological stability [2]. Effective pain management is critical to mitigate these risks [3].

Neonatal pain processing involves immature descending inhibitory pathways, making preterm infants particularly vulnerable to procedural pain [4]. Individual interventions—oral sucrose, maternal voice, and tactile stimulation—have shown variable efficacy in reducing neonatal pain during ROP screening [5]. Single‐modality approaches often fail to provide adequate pain relief during highly invasive procedures, with studies reporting inconsistent results [6].

The “3T sensory saturation method,” which integrates taste, auditory, and tactile stimulation, offers a comprehensive approach by engaging multiple sensory pathways to enhance comfort [5]. Previous research suggests that multimodal stimulation can effectively reduce procedural pain in infants [6].

This study investigates the efficacy of the 3T sensory saturation method during ROP screenings compared to conventional pacifier use. We hypothesized that the 3T method would improve comfort and reduce pain, as indicated by lower pain scores and better physiological markers such as higher oxygen saturation levels. Additionally, we aimed to identify risk factors for discomfort and develop a predictive model to optimize pain management strategies for these screenings. The findings could significantly enhance clinical practices and the overall experience for preterm infants undergoing ROP screening.

## 2. Methods

### 2.1. Study Population

This prospective, randomized controlled trial was conducted from April to August 2025 at our hospital. This study was approved by the Medical Ethics Committee of our hospital on March 29, 2025 (approval number: 202503016). Informed consent was obtained from parents or legal guardians of all participants.

The registration was completed after participant enrollment had commenced, which is acknowledged as a limitation of the study.

A total of 100 preterm infants meeting inclusion criteria were enrolled and randomly assigned using computer‐generated random numbers in a 1:1 ratio to either the 3T group or control group. Allocation concealment was maintained using sequentially numbered, opaque, sealed envelopes opened only after consent was obtained.

Inclusion Criteria: (1) gestational age between 32 and 36 weeks; (2) Apgar score ≥ 7 at birth; (3) birth weight between 1500 and 2500 g; (4) good sucking ability without vomiting or choking; (5) normal muscle tone and bilateral hearing test; (6) requirement for fundus screening Informed consent signed by family members or guardians; (7) absence of liver or kidney dysfunction; (8) no congenital genetic diseases; and (9) no congenital heart disease.

Exclusion Criteria: (1) Apgar score < 7 at 5 min after birth; (2) gastrointestinal malformations, necrotizing enterocolitis, or other organic gastrointestinal disease; (3) administration of medication affecting vital signs within 24 h before the procedure, such as sedatives or muscle relaxants; and (4) continuous pain stimuli or critically ill preterm infants with poor responsiveness, such as those with endotracheal intubation or nasal continuous positive airway pressure.

Rationale for Gestational Age Criteria: We focused on infants with GA 32–36 weeks because (1) this population represents a substantial proportion of ROP screening candidates with relatively stable hemodynamics, allowing for standardized intervention protocols; (2) infants < 32 weeks often require intensive respiratory support and have higher medical complexity, which could confound pain assessment; and (3) establishing efficacy in this moderately preterm population provides a foundation for future studies in more vulnerable infants < 32 weeks.

### 2.2. ROP Screening Procedure

All ROP examinations were performed by a single experienced pediatric ophthalmologist using indirect ophthalmoscopy with a 28‐diopter lens. The screening protocol followed American Academy of Pediatrics guidelines. Topical anesthesia consisted of 0.5% proparacaine hydrochloride eye drops administered 30 s before examination. A wire lid speculum was used to maintain eyelid opening, and scleral depression was performed as needed for peripheral retinal visualization. Each examination lasted approximately 3–5 min per eye.

### 2.3. Intervention Methods

 Conventional Management: Use of pacifiers during retinopathy screening.
 3T Sensory Saturation Method:
   ① Taste Stimulation: Administer 0.2–0.5 mL (4‐10 drops) of 25% glucose solution to the newborn 30 s before the retinopathy screening to induce sucking reflex. The glucose solution is delivered via a syringe connected to a pacifier.
   Auditory Stimulation: Play a prerecorded audio of the mother’s gentle, soothing voice from a departmental mobile phone placed 30 cm away from the newborn’s head, starting 30 s before the screening and continuing until 5 min after the screening.
   ② Tactile Stimulation: A nurse massages the newborn’s body using baby massage oil, ensuring the pressure does not cause changes in facial expression. The tactile stimulation starts 30 s before the screening and continues until the procedure ends. Typically, within 30 s, the newborn will focus on sucking, listening, and massage, exhibiting regular sucking movements (1 suck/second), at which point the retinopathy screening can proceed.

### 2.4. Outcome Measures and Blinding

Primary outcomes included pain scores (neonatal infant pain scale [NIPS]) and comfort scores (preterm infant comfort scale) assessed during ROP examination. Secondary outcomes included heart rate and oxygen saturation. All outcome assessments were performed by trained nurses who were blinded to group allocation. The assessors were not involved in the intervention delivery and were positioned to observe the infant without knowledge of which intervention had been administered. To maintain blinding, outcome assessors entered the room only after the intervention had been initiated and was ongoing; they were instructed to focus solely on the infant’s behavioral and physiological responses and to avoid directing attention toward the intervention being delivered. Assessors wore noise‐attenuating earplugs to minimize awareness of auditory stimulation, and a physical screen was placed between the assessor and the nurse administering the tactile stimulation, allowing the assessor an unobstructed view of the infant’s face, limbs, and monitoring parameters while limiting direct observation of the intervention procedures. Although complete blinding could not be guaranteed given the nature of multimodal sensory stimulation, these measures were implemented to minimize performance and detection bias as much as practically feasible.

### 2.5. Data Collection

1. Preterm Infant Data: Demographic and clinical characteristics
2. Maternal Data: Demographic and clinical characteristics
3. NIPS: Used to assess procedural pain in preterm and full‐term infants. The scale includes six behavioral indicators: facial expression, crying, breathing pattern, arm movements, leg movements, and state of arousal. Crying is scored on a 3‐point scale (0, 1, 2), while the other indicators are scored on a 2‐point scale (0 and 1). The total NIPS score ranges from 0 to 7, with higher scores indicating greater pain.
4. Preterm Infant Comfort Scale: The scale includes seven items: alertness, calm/agitated, respiratory status, physical activity, muscle tone, facial expression, and average heart rate. Each item is scored from 1 to 5, with 1 being good and 5 being poor. The total score ranges from 7 to 35, with higher scores indicating lower comfort. A total score > 21 indicates the need for intervention to reduce pain.

### 2.6. Statistical Analysis Protocol

All statistical tests were two‐sided, with *p* < 0.05 considered statistically significant. Data analysis was performed using R 4.1.0 Software. Continuous variables were expressed as mean ± standard deviation (mean ± SD), and categorical variables as frequencies and percentages. Independent sample *t*‐test was used for normally distributed variables and Mann–Whitney *U* test for non‐normally distributed variables. Normality was tested using the Shapiro–Wilk test. Chi‐square test or Fisher’s exact test for group comparisons.

Variables with *p* < 0.2 in univariate analysis were included in multivariate analysis. Two complementary approaches were used: (1) Multivariate linear regression to analyze continuous comfort scores, preserving full statistical power and (2) firth penalized logistic regression for binary outcomes (comfort score ≤ 21 vs. > 21) to address potential data separation issues common in small samples. A predictive model was constructed and visualized using a nomogram. Model performance was evaluated using receiver operating characteristic (ROC) curve analysis (area under the curve [AUC]) and calibration curves. Results were presented as odds ratios (OR) and 95% confidence intervals (CI). A predictive model was constructed based on multivariate logistic regression results and visualized using a nomogram. The model’s predictive performance was evaluated using ROC curve analysis, with the AUC calculated to measure discrimination ability. Calibration was assessed using calibration curves.

## 3. Results

### 3.1. Comparison of Baseline Demographic Data

A total of 100 preterm infants were included in this study, with 52 assigned to the 3T group and 48 to the control (CON) group. Baseline characteristics were well‐balanced between the two groups, with no statistically significant differences observed (Table 1). Specifically, infant characteristics including gender (*p* = 0.829), birth weight (*p* = 0.573), current weight (*p* = 0.797), gestational age (*p* = 0.430), postnatal age (*p* = 0.802), Apgar score (*p* = 0.590), and perinatal complications (*p* = 0.808) were comparable. Similarly, maternal data showed no significant differences in maternal age (*p* = 0.270), delivery mode (*p* = 0.760), conception method (*p* = 1.000), gravidity (*p* = 0.738), and parity (*p* = 0.130).

**TABLE 1:** Baseline characteristics and pre‐examination assessments.

Variable	3T group (*N* = 52)	Control group (*N* = 48)	*p* value
*Demographic Data*			
Gender (Male/Female)	26 (50.0%)/26 (50.0%)	22 (45.8%)/26 (54.2%)	0.829
Birth Weight (g)	2130 ± 268	2160 ± 275	0.573
Current Weight (g)	2188 ± 327	2203 ± 223	0.797
Gestational Age (weeks)	33.15 ± 2.55	32.71 ± 2.95	0.430
Postnatal Age (days)^∗^	6.92 ± 2.68	7.04 ± 2.02	0.802
Apgar Score	10 (10, 10)	10 (10, 10)	0.590
Maternal Age (years)	29.3 ± 2.79	29.9 ± 3.02	0.270
Delivery Mode (Cesarean/Vaginal)	31 (59.6%)	31 (64.6%)	0.760
	21 (40.4%)	17 (35.4%)	
Conception Method (ART/Natural)	11 (21.2%)	11 (22.9%)	1.000
	41 (78.8%)	37 (77.1%)	
Gravidity	3.54 ± 1.16	3.46 ± 1.22	0.738
Parity	2.17 ± 0.92	2.46 ± 0.94	0.130
Perinatal Complications (Yes/No)	13 (25.0%)	14 (29.2%)	0.808
	39 (75.0%)	34 (70.8%)	

*Pre-examination Vital Signs*			
Heart Rate (bpm)	144 ± 15.3	145 ± 13.3	0.653
SaO_2_ (%)	94.6 ± 2.84	94.9 ± 3.10	0.637

*Pre-examination NIPS Scores*			
Facial Expression	0.19 ± 0.40	0.12 ± 0.33	0.361
Crying	0.71 ± 0.78	0.56 ± 0.77	0.337
Breathing Pattern	0.23 ± 0.43	0.25 ± 0.44	0.824
Arm Posture	0.25 ± 0.44	0.15 ± 0.36	0.193
Leg Posture	0.19 ± 0.40	0.19 ± 0.39	0.952
Alertness State	0.15 ± 0.36	0.23 ± 0.42	0.346
NIPS Total Score	1.73 ± 1.33	1.50 ± 1.32	0.387

*Pre-examination Comfort Scale*			
Vigilance	2.60 ± 1.26	2.33 ± 1.12	0.271
Calm/Agitated	2.40 ± 0.85	2.60 ± 0.92	0.260
Respiratory Status	2.79 ± 0.64	2.77 ± 0.69	0.895
Physical Activity	3.27 ± 1.12	2.85 ± 1.17	0.073
Muscle Tone	3.46 ± 1.54	3.27 ± 1.50	0.532
Facial Expression	2.15 ± 1.11	2.46 ± 1.20	0.192
Mean Heart Rate	2.33 ± 0.98	2.21 ± 0.80	0.508
Total Score	19.0 ± 2.81	18.4 ± 2.80	0.301

*Note:* SaO_2_, oxygen saturation.

Abbreviations: ART, assisted reproductive technology; NIPS, neonatal infant pain scale.

^∗^The average postnatal age of 7 days reflects our institutional ROP screening protocol, which initiates screening earlier than some international guidelines to ensure timely detection in our high‐risk population. All infants met postmenstrual age criteria for ROP risk.

### 3.2. Comparison of Vital Signs and Pain Assessment Before Treatment

Prior to the examination, there were no significant differences in vital signs between the two groups, including heart rate (*p* = 0.653) and oxygen saturation (SaO_2_) (*p* = 0.637) (Table 1). Furthermore, baseline pain and comfort assessments revealed comparable conditions across both groups. The pre‐examination NIPS showed no significant differences in facial expression, crying, breathing pattern, arm posture, leg posture, alertness state, and the total NIPS score (all *p* > 0.05). Likewise, all items on the preterm infant comfort scale—including vigilance, calm/agitated state, respiratory status, physical activity, muscle tone, facial expression, mean heart rate, and total comfort score—exhibited no significant differences between the groups (all *p* > 0.05).

### 3.3. Comparison of Vital Signs and Pain Assessment During Treatment

During the examination, comparison of vital signs between the two groups showed no significant difference in heart rate (*p* = 0.0994), but SaO_2_ was significantly higher in the 3T group compared to the CON group (*p* < 0.0001) (Table 2). Pain assessment using NIPS showed that the 3T group had significantly lower scores in facial expression, crying, breathing pattern, alertness state, and total NIPS score compared to the CON group (*p* = 0.0199, *p* = 0.0162, *p* = 0.0293, *p* = 0.0302, and *p* < 0.0001, respectively). Differences in arm posture and leg posture were not significant (*p* = 0.0508 and *p* = 0.1247, respectively) . Comfort scale scores indicated that the 3T group had significantly lower scores in vigilance, calm/agitated, respiratory status, physical activity, muscle tone, mean heart rate, and total score compared to the CON group (all *p* < 0.0001), except for facial expression which showed no significant difference (0.9777) (Table 3).

**TABLE 2:** Vital signs and pain scores during ROP examination.

Variable	3T group (*N* = 52)	Control group (*N* = 48)	*p* value
*Vital Signs*			
Heart Rate (bpm)	174 ± 19.5	181 ± 19.3	0.099
SaO_2_ (%)	91.2 ± 2.84	85.8 ± 2.94	< 0.0001

*NIPS Scores*			
Facial Expression	0.13 ± 0.34	0.33 ± 0.48	0.020
Crying	0.21 ± 0.50	0.54 ± 0.80	0.016
Breathing Pattern	0.23 ± 0.43	0.44 ± 0.50	0.029
Arm Posture	0.27 ± 0.45	0.46 ± 0.50	0.051
Leg Posture	0.31 ± 0.47	0.46 ± 0.50	0.125
Alertness State	0.25 ± 0.44	0.46 ± 0.50	0.030
NIPS Total Score	1.40 ± 1.07	2.69 ± 1.45	< 0.0001

*Note:* SaO_2_, oxygen saturation.

Abbreviation: NIPS, neonatal infant pain scale.

**TABLE 3:** Comfort scale scores during ROP examination.

Variable	3T group (*N* = 52)	Control group (*N* = 48)	*p* value
Vigilance	2.13 ± 0.84	2.90 ± 0.59	< 0.0001
Calm/Agitated	1.90 ± 0.53	3.65 ± 0.81	< 0.0001
Respiratory Status	2.52 ± 0.54	3.77 ± 0.66	< 0.0001
Physical Activity	2.50 ± 0.67	3.38 ± 0.87	< 0.0001
Muscle Tone	2.85 ± 0.89	3.67 ± 1.02	< 0.0001
Facial Expression	3.44 ± 0.92	3.44 ± 0.80	0.978
Mean Heart Rate	2.87 ± 0.77	3.96 ± 0.80	< 0.0001
Total Score	18.2 ± 2.11	24.8 ± 2.20	< 0.0001

*Note:* Data are presented as mean ± standard deviation. Higher scores indicate lower comfort levels.

### 3.4. Risk Factor Analysis and Predictive Model for Analgesic Effect During Retinopathy Screening in Preterm Infants

Based on comfort scale scores during examination, infants were classified into adequate comfort (≤ 21 points, *n* = 68) and inadequate comfort (> 21 points, *n* = 32) groups.

**FIGURE 1:**
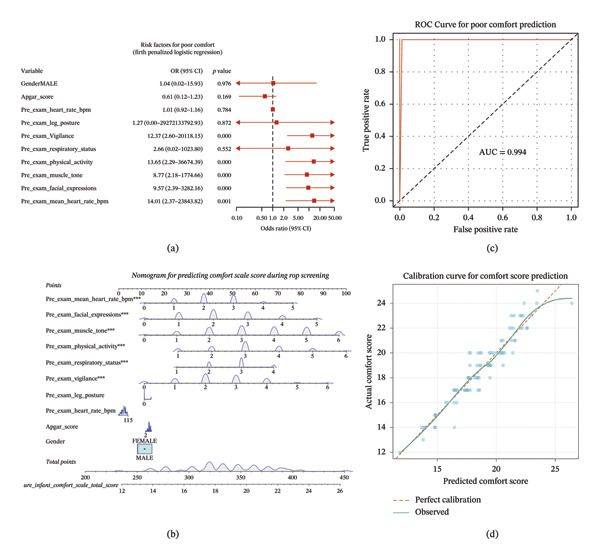
Risk Factor Analysis and Predictive Model for Analgesic Effect During Retinopathy Screening in Preterm Infants. (a) Forest plot showing multivariate regression analysis results; (b) nomogram showing the predictive model; (c) ROC curve; and (d) clinical calibration curve.

Univariate analysis identified 10 variables with *p* < 0.2 (Supporting Table 1). Multivariate linear regression (Table 4, Figure 1a) revealed that pre‐examination vigilance (*β* = 0.892, *p* < 0.0001), physical activity (*β* = 0.982, *p* < 0.0001), muscle tone (*β* = 0.947, *p* < 0.0001), facial expression (*β* = 1.007, *p* < 0.0001), and mean heart rate (*β* = 0.868, *p* < 0.0001) were significant predictors of comfort scores during screening.

**TABLE 4:** Multivariate linear regression analysis of comfort scores during examination.

Variable	Coefficient	SE	95% CI	*p* value
Gender (Male)	0.016	0.186	−0.354 to 0.385	0.933
Apgar Score	0.019	0.048	−0.077 to 0.114	0.702
Pre‐exam Heart Rate	−0.004	0.007	−0.017 to 0.009	0.530
Pre‐exam Leg Posture	0.194	0.241	−0.285 to 0.674	0.422
Pre‐exam Vigilance	0.892	0.079	0.735 to 1.049	< 0.0001
Pre‐exam Respiratory Status	0.944	0.139	0.668 to 1.220	< 0.0001
Pre‐exam Physical Activity	0.982	0.084	0.815 to 1.149	< 0.0001
Pre‐exam Muscle Tone	0.947	0.062	0.823 to 1.071	< 0.0001
Pre‐exam Facial Expression	1.007	0.081	0.847 to 1.168	< 0.0001
Pre‐exam Mean Heart Rate	0.868	0.106	0.658 to 1.078	< 0.0001

Firth penalized logistic regression (Table 5) confirmed these findings with clinically reasonable ORs: pre‐examination vigilance (OR = 12.37, 95% CI: 2.60–20118.15, *p* < 0.0001), physical activity (OR = 13.65, 95% CI: 2.29–36674.39, *p* = 0.0003), muscle tone (OR = 8.77, 95% CI: 2.18–1774.67, *p* = 0.0001), facial expression (OR = 9.57, 95% CI: 2.39–3282.16, *p* < 0.0001), and mean heart rate (OR = 14.01, 95% CI: 2.37–23843.82, *p* = 0.0007).

**TABLE 5:** Firth penalized logistic regression analysis for inadequate comfort (score > 21).

Variable	OR	95% CI	*p* value
Gender (Male)	1.040	0.021 to 15.934	0.976
Apgar Score	0.611	0.121 to 1.230	0.169
Pre‐exam Heart Rate	1.012	0.921 to 1.163	0.784
Pre‐exam Leg Posture	1.273	0.000 to 2.93 × 10^10^	0.872
Pre‐exam Vigilance	12.372	2.596 to 20118.15	< 0.0001
Pre‐exam Respiratory Status	2.664	0.024 to 1023.8	0.552
Pre‐exam Physical Activity	13.647	2.293 to 36674.39	0.0003
Pre‐exam Muscle Tone	8.774	2.177 to 1774.67	0.0001
Pre‐exam Facial Expression	9.570	2.390 to 3282.16	< 0.0001
Pre‐exam Mean Heart Rate	14.007	2.371 to 23843.82	0.0007

A nomogram was constructed based on these predictors (Figure 1b). The ROC curve demonstrated excellent discrimination with AUC = 0.994 (Figure 1c). The calibration curve showed good agreement between predicted and observed outcomes (Figure 1d).

## 4. Discussion

### 4.1. Efficacy of the 3T Sensory Saturation Method

The primary finding of this study is the superior efficacy of the 3T sensory saturation method compared to pacifier use alone in managing pain associated with ROP screening. During the examination, infants in the 3T group exhibited significantly higher oxygen saturation (SaO_2_) levels and lower NIPS scores across several key indicators, including facial expression, crying, arm posture, and alertness state. These results strongly suggest that the multimodal sensory stimulation provided by the 3T method effectively reduces pain perception in preterm infants. The observed improvement in physiological stability, as evidenced by the higher SaO_2_ levels, is particularly noteworthy [7]. Pain and distress can trigger physiological responses such as increased heart rate and decreased oxygen saturation, potentially leading to adverse outcomes in vulnerable preterm infants [8–10]. The 3T method appears to effectively counteract these responses, promoting a more stable and comfortable state during the screening procedure [11].

The comfort scale results further support the benefits of the 3T method. Infants in the 3T group demonstrated significantly lower comfort scale scores across multiple domains, including vigilance, calm/agitated, respiratory status, physical activity, muscle tone, and mean heart rate. This comprehensive improvement in comfort indicators suggests that the 3T method not only reduces pain but also promotes a state of relaxation and well‐being in preterm infants. The integration of taste (sweet solution), auditory (mother’s voice), and tactile (massage) stimulation likely works synergistically to saturate the infant’s sensory pathways, diverting attention from the painful stimulus and promoting a sense of security and comfort. This multimodal approach aligns with the understanding of neonatal pain processing, which emphasizes the importance of engaging multiple sensory modalities to effectively modulate pain perception.

### 4.2. Comparison With Existing Literature

Our findings are consistent with previous research demonstrating the effectiveness of nonpharmacological interventions for pain management in preterm infants. Studies have shown that sucrose administration, maternal voice, and tactile stimulation can individually reduce pain and distress during various procedures [12, 13]. The 3T method builds upon these individual interventions by combining them into a comprehensive multimodal approach. The synergistic effect of these stimuli may explain the superior outcomes observed in our study compared to studies using single interventions [11, 14].

However, it is important to acknowledge that some studies have reported inconsistent results regarding the effectiveness of nonpharmacological pain management techniques [15–17]. These inconsistencies may be due to variations in study design, sample characteristics, or the specific interventions used. Our study addresses some of these limitations by employing a standardized protocol for the 3T method and carefully controlling for potential confounding factors. Furthermore, we utilized validated pain and comfort assessment tools to objectively measure the impact of the intervention.

### 4.3. Clinical Implications and Future Directions

The findings of this study have significant implications for clinical practice. The 3T sensory saturation method offers a safe, effective, and readily implementable approach to pain management during ROP screening. Its ease of administration and low cost make it a practical alternative to pharmacological interventions, which may carry potential risks and side effects. Implementing the 3T method as a standard of care for ROP screening could significantly improve the experience of preterm infants and reduce the potential for adverse neurodevelopmental outcomes associated with unmanaged pain [16, 18, 19].

Furthermore, the development of a predictive model to identify infants who might benefit most from the 3T intervention has the potential to personalize pain management strategies [20, 21]. By identifying infants at higher risk for discomfort during ROP screening, clinicians can proactively implement the 3T method to optimize pain relief and promote comfort. Future research should focus on validating this predictive model in larger and more diverse populations.

Future research should also explore the long‐term effects of the 3T method on neurodevelopmental outcomes [22]. While our study demonstrated immediate benefits in terms of pain reduction and physiological stability, it is important to determine whether these benefits translate into improved long‐term outcomes. Additionally, further research is needed to optimize the components of the 3T method and identify the most effective combination of sensory stimuli. For example, future studies could investigate the optimal concentration of sucrose solution, the ideal volume of massage oil, or the most soothing characteristics of maternal voice recordings.

### 4.4. Predictive Model and Clinical Implications

Our predictive model identified pre‐examination behavioral and physiological markers that predict inadequate comfort during ROP screening. Infants with higher pre‐examination vigilance, physical activity, muscle tone, facial tension, and heart rate were at increased risk for discomfort during the procedure. This model (AUC = 0.994) could help clinicians identify high‐risk infants who would benefit most from enhanced pain management strategies like the 3T method.

The use of Firth penalized logistic regression addressed the statistical challenge of data separation in our sample, yielding more stable and clinically interpretable ORs compared to standard logistic regression. This methodological approach is particularly important in small‐sample neonatal studies where complete or quasi‐complete separation frequently occurs.

### 4.5. Limitations

Several limitations should be acknowledged. First, while outcome assessors were blinded, the nurses administering the interventions could not be blinded due to the nature of the 3T method, potentially introducing performance bias. Second, our sample was limited to moderately preterm infants (GA 32–36 weeks) with relatively stable clinical status; results may not generalize to extremely preterm infants (< 32 weeks) or those with significant comorbidities. Third, the single‐center design may limit external validity. Fourth, we assessed only immediate procedural outcomes; long‐term neurodevelopmental effects were not evaluated. Finally, our early screening protocol (average 7 days postnatal age) differs from some international guidelines, which may affect comparability with other studies.

## 5. Conclusion

In conclusion, this study provides compelling evidence for the effectiveness of the 3T sensory saturation method in mitigating pain and improving comfort in preterm infants undergoing ROP screening. The 3T method offers a safe, effective, and readily implementable alternative to pharmacological interventions and has the potential to significantly improve the experience of preterm infants. Future research should focus on validating these findings in larger and more diverse populations, exploring the long‐term effects of the 3T method on neurodevelopmental outcomes, and optimizing the components of the intervention.

## Author Contributions

Chaoqun Zhou: conceptualization, methodology, data curation, formal analysis, and writing–original draft. Xuejun Zhao: data curation, formal analysis, and writing–original draft. Jiamin Xu: data curation, investigation, visualization, and writing–review and editing. Yongli Ni: supervision, project administration, and writing–review and editing.

## Funding

The research did not receive funding from any sources.

## Disclosure

All authors have read and approved the final manuscript and agree with its submission.

## Ethics Statement

This study was approved by the Medical Ethics Committee of our hospital, with approval date: March 29, 2025, and ethics approval number: 202503016.

## Consent

Informed consent for participants under 18 years of age has been obtained from their parents or legal guardians.

## Conflicts of Interest

The authors declare no conflicts of interest.

## Supporting Information

Additional supporting information can be found online in the Supporting Information section.

## Supporting information


**Supporting Information** Table S1. Comparison of Baseline data.

## Data Availability

The datasets used and/or analyzed during the current study are available from the corresponding author on reasonable request.
