# Supplementary material for: Sensory Saturation (3T) for Pain Management During ROP Screening in Preterm Infants Born Between 32 to 36 Weeks of Gestational Age
Source: J Ophthalmol. 2026 Jun 3;2026:5427050. doi: 10.1155/joph/5427050 (PMC13239429; doi:10.1155/joph/5427050)
Supplement: Supplementary file 1 — Supporting Information Table S1. Comparison of Baseline data. [file JOPH-2026-5427050-s001.docx]

**Table S1. Comparison of Baseline data**

|  | **3T Group** | **Control Group** | **p.overall** |
| --- | --- | --- | --- |
|  | ***N=52*** | ***N=48*** |  |
| Group: |  |  | <0.0001 |
| 3T | 47(95.9%) | 3(5.88%) |  |
| CON | 2(4.08%) | 48(94.1%) |  |
| Gender: |  |  | 0.6891 |
| FEMALE | 26(53.1%) | 24(47.1%) |  |
| MALE | 23(46.9%) | 27(52.9%) |  |
| Birth_Weight_g | 2118±268 | 2170±288 | 0.3502 |
| Current_Weight_kg | 2200±332 | 2171±251 | 0.6269 |
| Gestational_Age_weeks | 35.6±3.85 | 34.9±3.56 | 0.3689 |
| Postnatal_Age_days | 7.08±2.57 | 6.59±2.06 | 0.2938 |
| Apgar_Score | 7.73±1.83 | 7.35±2.10 | 0.3344 |
| Maternal_Age_years | 29.3±2.80 | 29.6±2.91 | 0.5970 |
| Delivery_Mode: |  |  | 0.3243 |
| Cesarean | 29(59.2%) | 36(70.6%) |  |
| Vaginal | 20(40.8%) | 15(29.4%) |  |
| Conception_Method: |  |  | 1.0000 |
| ART | 11(22.4%) | 11(21.6%) |  |
| Natural | 38(77.6%) | 40(78.4%) |  |
| Gravidity | 3.24±1.56 | 3.04±1.56 | 0.5118 |
| Parity | 2.31±1.06 | 2.53±1.12 | 0.3092 |
| Perinatal_Complications: |  |  | 1.0000 |
| NO | 36(73.5%) | 37(72.5%) |  |
| YES | 13(26.5%) | 14(27.5%) |  |
| Heart_Rate_bpm | 145±14.8 | 145±14.4 | 0.8908 |
| SaO2_% | 94.7±2.94 | 94.8±3.11 | 0.9605 |
| Facial_Expression | 0.22±0.42 | 0.14±0.35 | 0.2628 |
| Crying | 0.76±0.80 | 0.55±0.76 | 0.1905 |
| Breathing_Pattern | 0.24±0.43 | 0.29±0.46 | 0.5835 |
| Arm_Posture | 0.22±0.42 | 0.20±0.40 | 0.7308 |
| Leg_Posture | 0.22±0.42 | 0.18±0.39 | 0.5539 |
| Alertness_State | 0.14±0.35 | 0.22±0.42 | 0.3468 |
| NIPS_Total_Score | 1.82±1.42 | 1.57±1.19 | 0.3483 |
| Vigilance | 2.55±1.24 | 2.27±1.20 | 0.2610 |
| Calm_Agitated | 2.43±0.84 | 2.69±0.79 | 0.1174 |
| Respiratory_Status | 2.76±0.66 | 2.78±0.70 | 0.8309 |
| Physical_Activity | 3.31±1.10 | 2.82±1.16 | 0.0356 |
| Muscle_Tone | 3.41±1.55 | 3.49±1.45 | 0.7855 |
| Facial_Expressions | 2.12±1.09 | 2.41±1.20 | 0.2107 |
| Mean_Heart_Rate_bpm | 2.29±0.94 | 2.25±0.89 | 0.8665 |
| Premature_Infant_Comfort_Scale_Total_Score | 18.9±2.84 | 18.6±2.76 | 0.7081 |
